# Unexpected Formation of Low Amounts of (*R*)-Configurated *anteiso*-Fatty Acids in Rumen Fluid Experiments

**DOI:** 10.1371/journal.pone.0170788

**Published:** 2017-01-27

**Authors:** Dorothee Eibler, Halima Abdurahman, Tanja Ruoff, Stefanie Kaffarnik, Herbert Steingass, Walter Vetter

**Affiliations:** 1 University of Hohenheim, Institute of Food Chemistry (170b), Stuttgart, Germany; 2 University of Hohenheim, Institute of Animal Science (460a), Stuttgart, Germany; Laval University, CANADA

## Abstract

*Anteiso*-fatty acids (*a*FA) with odd carbon number are a class of branched-chain fatty acids (BCFA) mainly produced by bacteria. Bacterial sources are also made responsible for their occurrence in the low percent-range in lipids of ruminants (meat and milk) and fish. *a*FAs are chiral molecules and typically occur predominantly in form of (*S*)-enantiomers, and their primary precursor has been noted to be isoleucine. Yet, low proportions of (*R*)-*a*FAs were also detected in fish and cheese samples. Here we investigated the potential formation of (*R*)-*a*FAs by means of incubation experiments with rumen fluid from fistulated cows. Supplementation of rumen fluid with both L- and DL-isoleucine, resulted in a significant (α <0.05) increase of the *a*FA concentrations but in both cases enantiopure (*S*)-*a*FAs were observed. By contrast, incubations without addition of any isoleucine lead to a significant (α <0.05) formation of small proportions of (*R*)-*a*FAs similarly to those previously observed in fish and cheese. These results were consistently reproduced in three different years with rumen fluid from different cows fed different diets. All findings point to the existence of a further biosynthesis pathway of *a*FAs with different stereospecificity than the classic one using isoleucine as primer.

## Introduction

*Anteiso*-fatty acids (*a*FA) are a class of branched-chain fatty acids (BCFA) which is characterized by a methyl-substituent located on the antepenultimate carbon of the carboxyalkyl chain [1,2]. The most relevant *a*FAs in food and bacteria are 12-methyltetradecanoic acid (*a*15:0) and 14-methylhexadecanoic acid (*a*17:0) [1,3,4]. Together with *iso*-fatty acids (*i*FAs; characterized by a methyl-substituent located on the penultimate carbon), *a*FAs are minor fatty acids in food [2,4–7], but the most prominent fatty acids of cell membranes of some bacteria, especially of the genus *Bacillus* [1,8–10]. These bacteria are utilizing BCFAs instead of unsaturated fatty acids for the modulation of the membrane fluidity [1,11,12].

**Fig 1:**
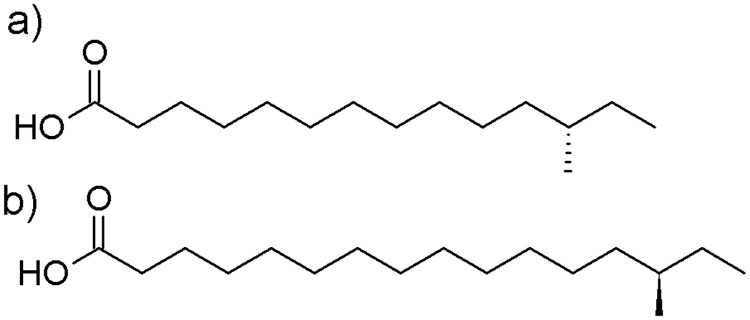
Chemical structure of (a) (*S*)-12-methyltetradecanoic acid (*a*15:0) and (b) (*R*)-14-methylhexadecanoic acid (*a*17:0).

Contrary to *i*FAs and classic fatty acids, *a*FAs are chiral molecules due to the stereogenic center at the carbon of the branching point (Fig 1) [1,8,13]. A distinct pre-dominance of (*S*)-*a*FAs or even (*S*)-enantiopurity has been described in fish [2,5,14], dairy products [2,5,15,16], mutton fat [17], Brussel sprouts [18] and bacteria [1,13,19]. However, two previous studies noted the occurrence of small amounts of (*R*)-enantiomers of *a*FAs in fish and cheese samples [2,5].

**Fig 2:**
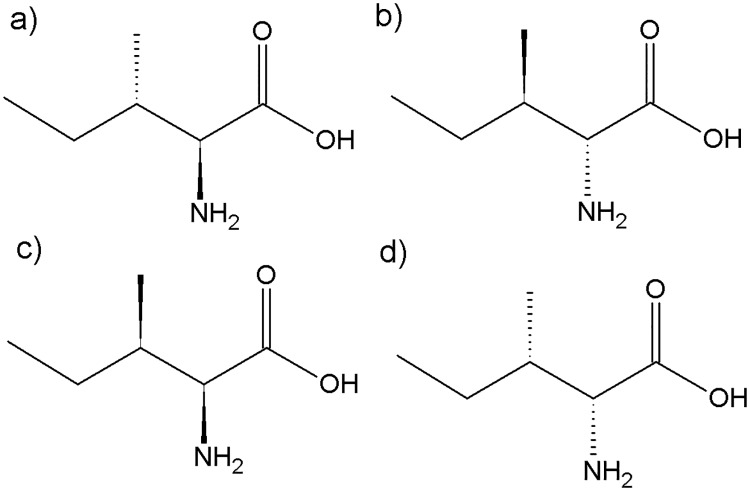
Chemical structures of the isomers of isoleucine (a) L-isoleucine ((2*S*,3*S*)-2-amino-3-methylpentanoic acid), (b) D-isoleucine ((2*R*,3*R*)-2-amino-3-methylpentanoic acid)) (c) L-*allo-*isoleucine ((2*S*,3*R*)-2-amino-3-methylpentanoic acid)) and (d) D-*allo*-isoleucine ((2*R*,3*S*)-2-amino-3-methylpentanoic acid).

Little knowledge existed about the stereoselective formation of *a*FAs and especially of (*R*)-*a*FAs. The most common primer in the biosynthesis of odd-numbered *a*FAs, 2-methylbutanoyl-CoA, is generated via transamination and decarboxylation of L-isoleucine (2*S*-amino-3*S*-methylpentanoic acid, L-ILE) [1]. In L-ILE (Fig 2a), the crucial stereocenter on C-3 is in (*S*)-configuration which, after activation and repeated chain elongation, leads to the stereospecific formation of (*S*)-*a*FAs [1,8,13]. However, this biosynthesis pathway cannot explain the presence of up to 10% (*R*)-*a*FAs in cheese and fish [2,5].

Next to the predominant L-ILE in nature (Fig 2a), stereoisomers are existing in form of D-*allo*-isoleucine (D-*allo*-ILE) (Fig 2d), D-isoleucine (D-ILE) (Fig 2b) and L-*allo*-isoleucine (L-*allo*-ILE) (Fig 2c), but these are occurring only in minute amounts on earth [20–22]. Kaneda observed an increase in (*S*)-*a*FA synthesis in *Bacillus subtilis* when L-ILE or L-*allo*-ILE were added to a medium but not with D-ILE and D-*allo*-ILE [13]. This was also found to be valid for rat skin [23]. Accordingly, L- (that is 2*S*-) configuration of the amino group on ILE was decisive for the biosynthesis of *a*FAs [8,13]. Using X-ray diffraction (XRD) measurements—after *a*FA enrichment by gas chromatographic fractionation—Kaneda found that both L-ILE and L-*allo*-ILE were able to generate (*S*)-*a*FAs [1,13]. In the case of L-*allo*-ILE this was unexpected because this pathway involved a conversion of the (*R*)-configuration on C-3 [13]. While it cannot be excluded that traces of (*R*)-*a*FAs would have been lost during the isolation procedure or overlooked during the measurements, enantioselective gas chromatography with mass spectrometry (GC/MS) analysis confirmed the sole presence of (*S*)-*a*17:0 in cultured *Bacillus megaterium* [19]. Hence, the biogenic formation of (*R*)-*a*FAs is still a mystery.

The aim of this study was to investigate the stereospecific formation of *a*FAs *in vitro* by means of incubation experiments with L-, L-*allo*- and racemic DL-ILE as well as without ILE. While several authors have studied *a*FA formation from ILE by pure or isolated bacterial strains [8,9,13,24–27], we chose to perform incubations with rumen fluid because this matrix is closer related to the milk fat in which (*R*)-*a*FAs had been previously detected. Comparably few attempts have been undertaken to explore the biosynthesis of fatty acids in this more complex microflora with symbiotic relationships [1,24,26,27]. Rumen fluid samples were taken from fistulated cows and incubated for 24 h. Thereafter, the quantities and relative contributions of *a*FAs to the fatty acid patterns were determined by GC/MS. The enantiomeric composition of *a*FAs as methyl esters was studied with enantioselective GC/MS [2,18]. To exclude random results caused by special conditions due to the microorganisms in rumen fluid the experiment was repeated with different cows in three years. All data produced in these three years will be present without omissions.

## Materials and Methods

### Chemicals and standards

Tetradecanoic acid ethyl ester, 10,11-dichloroundecanoic acid, enantiopure (*S*)-*a*15:0 and (*S*)-*a*17:0 were synthesized by Thurnhofer *et al*. [28,29]. Racemic *a*FA standards (*a*13:0, *a*14:0, *a*15:0, *a*16:0, *a*17:0, *a*18:0, *a*19:0) and *i*FAs (*i*14:0, *i*15:0, *i*16:0, *i*17:0, *i*18:0) were from Larodan (Malmö, Sweden). A Supelco 37-component fatty acid methyl ester mix, DL-ILE (99% pure), L-*allo*-ILE (pure) and L-ILE (>98% pure) were from Sigma-Aldrich (Taufkirchen, Germany). Ethyl acetate, cyclohexane (both purest), methanol and *n-*hexane (both HPLC grade, Chem Solute) were ordered from Th. Geyer (Renningen, Germany). Ethyl acetate/cyclohexane and methanol/ethyl acetate were combined (1:1, v/v) and distilled to give the corresponding azeotropes. Chloroform (HPLC grade) was from VWR (Fontenay-sous-Boia, France), while bulk sorbent (isolute-HM-N) was from Biotage (Uppsala, Sweden). Boron trifluoride reagent (13–15% in methanol) was from Sigma Aldrich (Steinheim, Germany). Sodium chloride (99.8%), potassium hydroxide (min. 85% pure), sulfuric acid (Rotipuran, 98%) and urea (>99.5% pure) were from Carl Roth (Karlsruhe, Germany). Helium (5.0 quality) was from Westfalen (Münster, Germany). The carbohydrate mixture (CHO mixture) was composed of corn starch (Roquette, Frankfurt/Main, Germany), cellulose (Rettenmaier, Rosenberg, Germany) and sucrose (Sigma Aldrich, Taufkirchen, Germany) (50:30:20, w/w/w). Salts for the incubations (Na_2_HPO_4_ (>99% pure), KH_2_PO_4_ (>98% pure), MgSO_4_ x 7 H_2_O (>99.5% pure), CaCl_2_ x 2 H_2_O (>97% pure), MnCl_2_ x 4 H_2_O (>99% pure), CoCl_2_ x 6 H_2_O (>98% pure), FeCl_3_ x 6 H_2_O (>99% pure), NaHCO_3_ (>98% pure), NH_4_HCO_3_ (>98% pure), NaOH (purest) were from Merck (Darmstadt, Germany). Resazurin-Na-salt (analytical grade) was from Serva (Heidelberg, Germany), while Na_2_S x H_2_O was from Fluka (St. Gallen, Switzerland).

### Solutions for the incubations

All solutions for the incubation procedure were prepared according to Menke *et al*. [30]. The macro-mineral solution for the incubation experiments contained Na_2_HPO_4_ (5.7 g/L), KH_2_PO_4_ (6.2 g/L) and MgSO_4_ x 7 H_2_O (0.6 g/L), diluted in demineralized (demin.) water. The micro-mineral solution consisted of 13.2 g CaCl_2_ x 2 H_2_O, 10.0 g MnCl_2_ x 4 H_2_O, 1.0 g CoCl_2_ x 6 H_2_O and 8.0 g FeCl_3_ x 6 H_2_O in 100 mL demin. water. The buffer solution was obtained by dissolving 35 g NaHCO_3_ and 4 g NH_4_HCO_3_ in 1 L demin. water. The medium consisted of 155 mL demin. water, 0.04 mL micro-mineral solution, 77.5 mL buffer, 77.5 mL macro-mineral solution, and 0.4 mL of a resazurin solution (1 mg resazurin/1 mL demin. water). The reduction-solution contained 15.5 mL demin. water, 0.65 mL of a 1 M NaOH solution and 93.3 mg Na_2_S x H_2_O.

### Rumen fluid

Rumen fluid was taken from two rumen-fistulated non-lactating Holstein cows (February 2010) and two lactating Jersey cows (May 2011 and April 2015) (*Bos primigenius Taurus* L., 1758) held by the Institute of Animal Science (460a) at University of Hohenheim, Emil-Wolff-Str. 10, 70599 Stuttgart, Germany. Holstein cows were fed hay (80%) and concentrates (20%) (in total 10 kg feeding stuff) while Jersey cows obtained 65% forage based on hay, grass and corn silage, and 35% concentrated feed. In all years, the concentrate contained grain, grain legumes as well as rape expeller in the same ratio. Cows were fed twice a day at 8 a.m. and 4 p.m. Rumen fluid was collected from the cows prior to the morning feeding into pre-warmed thermos flasks. The rumen fluid from both cows was mixed approximately 1:1 and filtered through two layers of cheese cloth to separate coarse feed particles. All animal studies reported herein were in accordance with the animal welfare legislation and approved by the District Council of Stuttgart, Germany.

### Incubation procedure

Medium (310 mL) was warmed up to 39°C with a water bath in a three-necked flask and reduction solution (16 mL) was added under CO_2_ flushing and gently agitated with a magnetic stirrer. After the color of the mixture had changed from blue over red to colorless (~15 min), 162.5 mL rumen fluid was added and CO_2_ flushing was continued for 15 min [30]. Aliquots of the incubation solution (30 mL) were transferred under CO_2_ flushing and stirring into incubation flasks (50 mL, Sarstedt, Nümbrecht, Germany) containing all ~200 mg CHO mixture (see above). Four (2010, 2015) or seven (2011) different substrate treatments were processed in duplicate in each incubation experiment (S1 Table). Treatment 1 contained ~13 mg L-ILE and treatment 2 ~13 mg DL-ILE. Treatment 3 which did not contain ILE and treatment 4 which was supplemented with ~6 mg urea instead of ILE served as controls (S1 Table). Treatments were generally performed in duplicate in the years 2010, 2011 and 2015. In 2011, additional experiments (in duplicate) were performed named treatment 5 with L-*allo*-ILE (~12 mg) plus urea (~7.4 mg) and treatment 6 with both L-ILE (~12 mg) and urea (~7.4 mg). For these incubations, one sample with ~200 mg CHO mixture and ~ 10.4 mg urea served as control (treatment 7, corresponding to treatment 4, with varied amounts of supplementation) (S1 Table). After mixing, incubations were executed in a shaking water bath (39°C) anaerobically by means of a Bunsen valve and stopped after 24 h by freezing the samples on ice. Incubations for 24 h have been classified as representative for feeding stuffs with an average retention time in the rumen and an average passage rate [30]. Therefore, this setup seemed to represent the rumen biology to an appropriate degree.

### Primary note

The experiments in this studies lasted for five years. During this period the lipid extraction method and the transesterification procedure was modified in our laboratory, and different methods were used in 2010/2011 and 2015 which, however, led to the same results. In 2010 and 2011, lipids were extracted by accelerated solvent extraction (for details see below). Since this instrument was not available for the third study in 2015, lipids were extracted using the method of Bligh and Dyer (for details see below) [31]. Initial analysis of a rumen fluid sample (n = 4) verified the good agreement of the lipid extraction by accelerated solvent extraction (1.08% lipids) and extraction according to Bligh and Dyer (1.12% lipids). Likewise, the fatty acid pattern varied less than 1% of each fatty acid´s contribution to the total fatty acids, and the enantiomeric excess of *anteiso*-fatty acids was also the same. Likewise, the formation of fatty acid methyl esters was different in 2015. In either case we used official standard methods suggested by the German Society of Fat Research (DGF) [32]. Initial comparison of both transesterification methods by means of a rumen fluid sample (n = 6) resulted in very similar results (amount of each fatty acid varied less than 1%) and standard deviations of both methods (0.46 for BF_3_ and 0.41 for sulfuric acid method, mean value of standard deviations calculated for all detectable fatty acids) showed a comparable robustness of both methods.

### Lipid extraction

Samples were frozen and lyophilized for five days in a LYOVAC GT 2 system (Leybold-Heraeus, Hürth, Germany) at 0.1 mbar.

*Samples from 2010 and 2011*. Lipids were gained by accelerated solvent extraction (ASE 200, Dionex, Idstein, Germany) [3]. In brief, ~1 g lyophilized sample was placed in 22 mL extraction cells and then filled to the brim with bulk isolute sorbent. Extraction was performed first with 2x 40 mL of ethyl acetate/cyclohexane (1:1 v/v) and second with 2x 40 mL of methanol/ethyl acetate (1:1 v/v) (125°C, 10 MPa, heating time 6 min). The combined extracts were concentrated in a rotary evaporator (200 mbar, 35°C bath temperature) and adjusted to exactly 5 mL in a calibrated flask. Aliquots (1 mL) were used for gravimetric determination of the lipid content.

*Samples from 2015*. Lipids were extracted according to the method of Bligh and Dyer [31]. For this purpose lyophilized samples were weighed into incubation flasks and supplemented with 16 mL bidistilled water, 20 mL chloroform and 40 mL methanol. Sample homogenization was carried out with an turbo-powered hand-held blender (Ultra Turrax T25, Janke & Kunkel IKA Labortechnik, Staufen i.Br., Germany), operated at 10,000 rpm for 2 min. After sample transfer into a graduated cylinder, 20 mL chloroform was added and the mixture was shaken for 30 s. Bidistilled water (20 mL) was added and shaking was continued for another 30 s. After phase separation, the lower chloroform-phase was separated and filtered through a folded filter (185 mm diameter; Schleicher & Schuell, Dassel, Germany). The residue was washed with ~5 mL chloroform. Then, the filtrate was evaporated and the volume adjusted to 5 mL. One milliliter was used for the gravimetric lipid determination. The dry matter content of the samples was about 1.4% and the lipid content of the dry matter was about 1.1%.

### Preparation of fatty acid methyl esters

After lyophilizing and lipid extraction, the samples were aliquoted and each was transferred into FAMEs and analyzed in duplicate. Hence, four samples were obtained for each treatment.

*Samples from 2010 and 2011*. Transesterification was prepared according to DGF Standard Method C-VI a [32]. Aliquots of ASE extracts (twice per each sample, therefore four samples for each treatment) (representing ~1 mg fat) were evaporated to dryness, 0.5 mL 0.5 M methanolic KOH was added and the samples were placed in a sand bath (80°C, 5 min) for hydrolysis. After cooling on ice, the free fatty acids were methylated in a sand bath (80°C, 5 min) by adding 1 mL of boron trifluoride/methanol solution. The ice-cooled samples were mixed with 2 mL pure water and saturated aqueous solution of NaCl and FAMEs were extracted by adding 1 mL *n*-hexane. After dilution (c_FAME_ ~0.2 mg/mL) the samples were analyzed by GC/MS.

*Samples from 2015*. FAMEs were generated according to DGF Standard Method C-VI f [32] and quantification was performed according to the method of Thurnhofer *et al*. [29]. In brief, 10 μL 10,11-dichloroundecanoic acid solution (internal standard I, 1 mg/mL) was added to ~1.5 mg dry matter of the chloroform extracts (twice per each sample, therefore four samples for each treatment) and methylated with 2 mL 1% sulfuric acid in methanol in a sand bath (80°C, 1.5 h). After cooling on ice, 2 mL pure water and 2 mL saturated aqueous solution of NaCl were added. Extraction of FAMEs was performed with 2 mL *n*-hexane (c_FAME_ ~0.75 mg/mL). One fourth of this solution (500 μL) was diluted with 500 μL *n*-hexane (c_FAME_ ~0.375 mg/mL) and 10 μL tetradecanoic acid ethyl ester solution (internal standard II, 0.5 mg/mL) was added.

For each treatment the standard deviation was calculated. Due to the excellent standard deviation values (deviations < 1%), only mean values will be discussed in the text, while error bars as well as the standard deviation are shown in tables and figures. Fatty acids were measured as methyl esters but will be named fatty acids only in section Results and Discussions in order to keep the text simpler.

### Gas chromatography with electron ionization mass spectrometry (GC/MS)

Non-enantioselective analyses were carried out with an HP 5890 series II gas chromatograph equipped with an HP 7673A autosampler and an HP 5971A mass spectrometer operated at 70 eV (Hewlett-Packard/Agilent, Waldbronn, Germany). Splitless injections (1 μL injected) were conducted at 250°C. A 60 m x 0.22 mm internal diameter fused silica column coated with 0.1 μm film thickness of 10% cyanopropylphenyl, 90% *bis*-cyanopropyl polysiloxane (Rtx-2330, Restek, Bellefonte, USA) was installed in the GC oven. Helium was used as the carrier gas with a constant flow rate of 1 mL/min. The GC oven temperature started at 60°C (hold for 1 min). It followed heating ramps of 6°C/min to 150°C, of 4°C/min to 190°C and of 7°C/min to 250°C (hold for 7 min) (modified from Thurnhofer *et al*.) [18,33]. The transfer line and the ion source temperatures were set at 280 and 165°C. After a solvent delay of 8 min, *m/z* 50–550 was measured in the full scan mode. In the selected ion monitoring (SIM) mode, *m/z* values representing the molecular ions of different FAMEs were recorded in nine time windows [18]: (i) 17.0–20.0 min: *m/z* 228 (isomers of 13:0-ME) and *m/z* 242 (isomers of 14:0-ME); (ii) 20.0–21.5 min: *m/z* 240 (isomers of 14:1-ME) and *m/z* 256 (isomers of 15:0-ME); (iii) 21.5–23.0 min: *m/z* 254 (isomers of 15:1-ME) and *m/z* 270 (isomers of 16:0-ME); (iv) 23.0–24.5 min: *m/z* 268 (isomers of 16:1-ME) and *m/z* 284 (isomers of 17:0-ME); (v) 24.5–25.9 min: *m/z* 282 (isomers of 17:1-ME) and *m/z* 298 (isomers of 18:0-ME); (vi) 25.9–26.6 min: *m/z* 296 (isomers of 18:1-ME) and *m/z* 312 (isomers of 19:0-ME); (vii) 26.6–29.2 min: *m/z* 294 (isomers of 18:2-ME) and *m/z* 292 (isomers of 18:3-ME); (viii) 29.2–31.5 min: *m/z* 320 (isomers of 20:3-ME) and *m/z* 340 (isomers of 21:0-ME); (ix) 31.5 min-41.5 min: *m/z* 368 (isomers of 23:0-ME). In addition, *m/z* 74, *m/z* 87, *m/z* 81, *m/z* 79, *m/z* 88 and *m/z* 101 were measured in all time windows throughout the run.

Enantioselective GC/MS analyses were performed with an HP GCD plus system equipped with an HP 6890 autosampler (Hewlett-Packard, Waldbronn, Germany). For injections in splitless mode the injection port was heated to 250°C. A 30 m x 0.25 mm internal diameter capillary column coated with 66% *heptakis*(2,3-di-*O*-methyl-6-*O*-*tert*-butyldimethylsilyl)-β-cyclodextrin (β-TBDM) (0.18 μm film thickness) in OV-1701 (custom-made by Georg Hottinger, BGB Analytik, Adliswil, Switzerland) was installed in the GC oven. The ion source (operated with 70 eV) and transfer line temperatures were set to 280°C and 165°C, respectively. The carrier gas helium was used with a flow rate of 1.0 mL/min. GC/MS-SIM runs were based on recording fragment ions characteristic for fatty acid methyl esters, i.e. *m/z* 74, *m/z* 87 along with the molecular ions *m/z* 228 (M^+^ of 13:0-ME isomers), *m/z* 242 (M^+^ of 14:0-ME isomers), *m/z* 256 (M^+^ of 15:0-ME isomers including *a*15:0-ME), *m/z* 270 (M^+^ of 16:0-ME isomers) and *m/z* 284 (M^+^ of 17:0-ME isomers including *a*17:0-ME) throughout the run. GC runs exceeding the maximum run time of 650 min programmable with the method software were conducted by combination of two subsequent runs in which the sample was injected in run-1 which lasted for 650 min. With the equilibration time set to 0 s run-2 was started immediately with the final temperature of run-1 with the injection of air and data recording without solvent delay [2,18]. In brief, the oven program of run-1 started with 60°C, held for 1 min, followed by a ramp of 20°C/min to 112°C (held for 455 min) and ramp of 1°C/min to 132°C (held for 171 min) (run time 650 min). Run-2 started at 132°C (held for another 260 min), raised to 200°C with a ramp of 30°C/min and held for 10 min, to elute associated compounds in the samples [2,18].

Small peaks in GC/MS-SIM chromatograms (*m/z* 74 and *m/z* 87) of samples were smoothed using the method of Savitzky and Golay [2,18,34]. Results were expressed by the enantiomeric excess (*ee* in %) of the (*S*)-enantiomers according to eq 1 [35]:

$$ee=\;\frac{\left(S\right)-\left(R\right)}{\left(S\right)+\left(R\right)}\;\cdot \;100\;\left[\%\right]$$ (1)

with (*S*) and (*R*) being the concentrations of the (*S*)- and (*R*)-enantiomer of the methyl ester of *a*13:0, *a*15:0 and *a*17:0, respectively.

Based on literature data, we expected a distinct dominance of (*S*)-*a*FAs and possible traces of the earlier eluting (*R*)-enantiomers [1,2,5,13,15,16,18], whereas a 100% enantiomer resolution could not be achieved on the β-TBDM column Therefore, evaluation of *ee* in sample solutions was carried out with enantiopure (*S*)-*a*15:0-ME and (*S*)-*a*17:0-ME and mixtures of them with racemic *a*15:0-ME and *a*17:0-ME to give 2% (*ee* = 96%), 5% (*ee* = 90%), 10% (*ee* = 80%) and 25% (*ee* = 50%) of (*R*)-*a*FAME in the corresponding solutions [18]. From the resulting GC/MS chromatograms (after smoothing) it was determined that *ee* = 98% was the maximum value for *a*15:0-ME that could be distinguished from (*S*)-enantiopurity under these chromatographic conditions and *ee* = 96% was the corresponding limit for *a*17:0-ME. Because of the lack of an enantiopure standard of *a*13:0-ME, the *ee* could only be calculated for *a*15:0-ME and *a*17:0-ME. GC/MS-SIM chromatograms of sample solutions were compared with standards by means of *m/z* 74 and *m/z* 87. Enantiopurity of *a*FAs (*ee* > 98% for *a*15:0-ME and >96% for *a*17:0-ME) was defined, when no deviation in the peak shape was noticeable between the neat (*S*)-*a*FAME standard and the corresponding peak in the sample solution. If (*R*)-enantiomers were detected, *ee* was assigned on basis of the closest match of the abundance of the shoulder peak with one of the four non-racemic reference standards [18].

### Statistical analyses

Differences between concentrations and ratio of Σ*a*FA/Σ*i*FA observed in incubations with different treatments within one year were evaluated by use of the unpaired t-test after verifying a statistical normal distribution [36]. Variances (α-value was <0.025) were homogenous in all cases [36]. Comparably, the evaluation of the statistical significance of enantioselective measurements was performed by use of the absolute amounts of each (*R*)- and (*S*)-*a*FAs within each treatment and taking the LOD into account for enantiopure (*ee* > 98% for *a*15:0-ME and >96% for *a*17:0-ME) samples. Homogeneity test for comparable variances was two-sided, while the t-test was performed both one-sided and two-sided. α -values of <0.05 were considered significant [36].

## Results and Discussion

### BCFAs in rumen fluid samples

Non-incubated rumen fluid samples contained three odd-numbered *a*FAs (*a*15:0 > *a*17:0 > *a*13:0, Table 1) and six *i*FAs (*i*13:0-*i*18:0) (S2 Table). *i*15:0 and/or *i*16:0 were dominant and represented >50% of the *i*FAs (S2 Table). The sum of *a*FAs (*i*FAs) was between 38–41 (36–38) mg/g fat (3.6–3.8% of the total fatty acids) (Table 1). The BCFA pattern was similar to the one described for *Bacillus* species and mixed rumen bacteria (dominance of *i*FAs and odd-numbered *a*FAs in the range of 14 to 17 carbons with highest individual contributions by *a*15:0) [10,37,38]. The moderate differences between the BCFA patterns in 2011 and 2015 was considered normal for rumen fluids from different animals fed different diets (Table 1).

**Table 1:** Concentrations including the standard deviation (mg/g fat) of *anteiso*-fatty acids, corresponding concentrations of (*R*)- and (*S*)-enantiomers as well as the sum concentration of *iso*-fatty acids in rumen fluid before (non-incubated) and after incubation with carbohydrates only or with carbohydrates and urea, L-ILE or DL-ILE. Data are means ± standard deviation of four samples.

	Carbohydrates only			Carbohydrates and urea			Carbohydrates and L-ILE			Carbohydrates and DL-ILE			Non-incubated rumen fluid	
	2015	2011	2010	2015	2011	2010	2015	2011	2010	2015	2011	2010	2015	2011
**mg/g fat**														
*a*13:0	2.53±0.043	1.20±0.012	1.50±0.14	2.03±0.22	2.20±0.14	0.50±0.12	2.53±0.099	2.20±0.071	1.00±0.26	3.51±0.14	4.10±0.35	3.00±0.01	0.70±0.01	0.60±0.01
							(+0%)*	(+35%)*	(-33%)*	(+38%)*	(+242%)*	(+100%)*		
*a*15:0	34.9±0.28	24.6±0.49	45.5±0.42	28.7±0.43	27.5±0.85	44.0±0.84	49.9±0.14	34.9±0.98	51.0±0.90	47.6±0.42	37.7±0.56	100±1.30	29.0±0.14	31.0±0.14
							(+43%)*	(+42%)*	(+12%)*	(+36%)*	(+53%)*	(+120%)*		
*(R)-a*15:0	1.75	1.23	2.28	2.87	2.75	4.40	n.d.	n.d.	n.d.	n.d.	n.d.	n.d.	n.d.	n.d.
*(S)-a*15:0	33.1	23.4	43.2	25.8	24.8	39.6	49.9	34.9	51.0	47.6	37.7	100	29.0	31.0
*a*17:0	6.98±0.035	11.3±0.14	14.5±0.56	4.60±0.86	10.8±0.49	14.5±0.14	8.82±0.39	15.6±0.98	14.5±0.98	9.38±0.24	20.9±0.98	63.0±0.91	8.00±0.14	9.50±0.56
							(+26%)*	(+38%)*	(+0%)*	(+34%)*	(+85%)*	(+335%)*		
*(R)-a*17:0	0.69			0.23			n.d.	n.d.	n.d.	n.d.	n.d.	n.d.	n.d.	n.d.
*(S)-a*17:0	6.28			4.37			8.82	15.6	14.5	9.38	20.9	63.0	8.00	9.50
**Σ *aFAs***	**44.4±0.27**	**37.1±0.35**	**61.5±0.70**	**35.4±1.50**	**40.5±1.55**	**59.0±0.021**	**61.3±0.35**	**52.7±1.20**	**66.5±1.60**	**60.5±0.91**	**62.7±1.20**	**166±1.02**	**37.7±0.28**	**41.1±0.71**
							(+38%)*	(+42%)*	(+8%)*	(+36%)*	(+69%)*	(+170%)*		
**Σ *iFAs***	**61.9±0.14**	**42.6±0.71**	**43.1±0.14**	**42.2±0.86**	**49.9±1.45**	**47.5±0.71**	**42.5±0.71**	**32.1±0.71**	**42.2±0.96**	**56.4±0.53**	**38.0±1.01**	**50.1±1.20**	**36.3±0.42**	**38.2±1.90**

* percentage growth or decrease in relation to the amount in incubations with carbohydrates only

**Fig 3:**
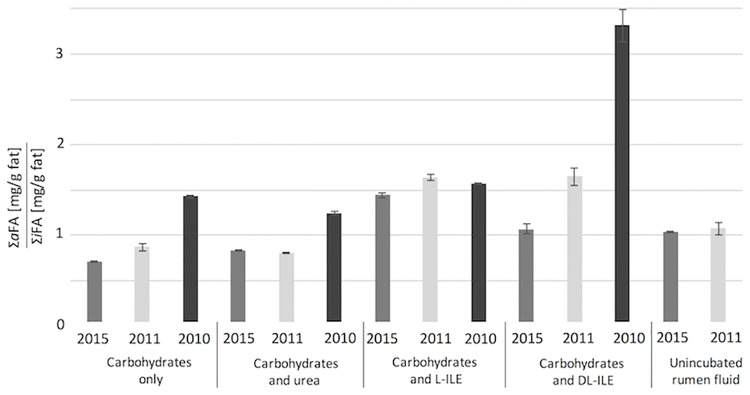
Concentration ratio of *anteiso*-fatty acids (∑*a*FAs [mg/g fat]) to *iso*-fatty acids (∑*i*FAs [mg/g fat]) including error bars in rumen fluid samples in the years 2015, 2011 and 2010. Significant differences between the ratio ∑*a*FAs /∑*i*FAs of unincubated rumen fluid and incubated ones as well as between the control treatment, Carbohydrates only” and between other treatments were found by use of the t-test with a statistical significance of α< 0.01.

Most incubation treatments slightly increased the concentration of *i*FAs in the samples compared to non-incubated rumen fluid, but there was no clear difference between treatments with ILE (L-ILE or DL-ILE) and without ILE (treatment with CHO-mixture and CHO-mixture plus urea) within all years (S1 and S2 Tables). A significant change (α < 0.05) in *a*FA concentrations (mostly an increase) was also noticed for most treatments, especially in those with ILE (Table 1). These results verify microbial activity during the incubations. Therefore, the effect of ILE supplementation was evaluated in comparison to results of incubations without ILE (carbohydrates only). The significant increase (α < 0.05) in the sum of *a*FA concentrations in all ILE treatments (between +8% and +170%, Table 1) verified that a varied share of ILE had been utilized by the microbial community in the rumen fluid. This can also be seen from the significant (α < 0.05) higher ratio of Σ*a*FAs to Σ*i*FAs in incubations with L- or DL-ILE, albeit the magnitude varied from year to year (Fig 3). Significant increasing concentrations were found for both *a*15:0 and *a*17:0 (with exception in 2010, L-Ile), while the concentration ratio between both *a*FAs remained almost constant in most occasions. This evaluation, verified in three different years, showed that L-ILE supplemented to rumen juice could be utilized by rumen microorganisms to produce *a*FAs [1]. Increasing amounts of *a*15:0 had also been observed with addition of L-ILE to *Bacillus* species [13,24]. Likewise, radiolabeled L-ILE was used in *in vitro* experiments with mixed rumen protozoa to show that it was preferably incorporated in branched-chain 15:0 and 17:0 compared to branched-chain 13:0 [38]. Although not specifically mentioned by Harfoot [38] it is likely that this increase was also due to the formation of *a*FAs and not of *i*FAs. Similarly, L-ILE intake via the feed correlated with the *a*FA amount in both skin lipids of rats [23,39] and blubber of a marine whale (*Stenella caeruleo alba*) [40].

Oku *et al*. reported that L-ILE and twice the amount of DL-ILE generated the same quantity of *a*FAs by exploring the biosynthesis of BCFA in rat skin [23]. In our study, increases in *a*FA concentrations were observed for both L-ILE and DL-ILE, without clear differences. Most likely, there was an excess of ILE in our incubation solutions with only L-ILE being utilized in both treatments. This hypothesis, based on the assumption that L-ILE was the principal precursor of *a*FAs, was examined by enantioselective analysis.

### Enantioselective analysis of *anteiso*-fatty acids

(*S*)-Enantiopure *a*FAs were detected in all rumen fluid samples (p = 1) before the incubations (Table 2). Likewise, all treatments with L- or DL-ILE only contained (*S*)-enantiomers of *a*13:0, *a*15:0 and *a*17:0 (p = 1,Table 2). This verified that only L-ILE (and not D-ILE) was utilized to generate the newly formed share of *a*FAs. Enantiopure (*S*)-*a*17:0 had also been observed in solutions with B. *subtilis* when grown on both standard culture and supplemented with DL-α-methyl butyrate [8,13,41].

**Table 2:** Enantiomeric excess (*ee*) [%] of *anteiso*-fatty acids in rumen fluid before (non-incubated) and after incubation with carbohydrates only as well as with carbohydrates and urea, L-ILE, DL-ILE or L-*allo*-ILE. Data are means of four samples. No standard deviation was calculated because of the same calculated ee within these measurements.

	Carbohydrate only			Carbohydrate and urea			Carbohydrate and L-ILE			Carbohydrate and DL-ILE			Carbohydrate and L-*allo*-ILE	Non-incubated rumen fluid	
year	2015	2011	2010	2015	2011^#^	2010	2015	2011	2010	2015	2011	2010	2011	2015	2011
**ee [%]**															
*a*13:0	<100***	*	*	<100***	*	*	100	*	*	100	*	*	*	100	*
*a*15:0	90	90	90	80	80	<90	100	100	100	100	100	100	80	100	100
*a*17:0	80	<100**	<100**	90	<100**	<100**	100	100	100	100	100	100	90	100	100

* not calculated due to an interference

** no quantitative calculation of the ee because of insufficient enantioseparation

*** no quantitative calculation of the ee because no (*S*)-pure standard available

^#^ same results in both control incubations with different urea amounts in 2011

To our surprise, rumen fluid samples incubated without addition of ILE (basically performed as controls) varied significantly (α < 0.05) and contained between 5 and 10% (*R*)-*a*15:0 and (*R*)-*a*17:0 (*ee* = 80–90%, Table 2). The presence of (*R*)-*a*15:0 and (*R*)-*a*17:0 was verified without exception (p = 1) in all rumen fluid incubations with three different rumen fluids in three different years (2010, 2011 and 2015) (see Materials and Methods). Enantioselectivity of *a*13:0 was only studied in 2015. Treatments without L-ILE were characterized by a small but distinct shoulder fronting the peak of *a*13:0 (indicative for the presence of (*R*)-*a*13:0) which was not observed in treatments with L-ILE.

The presence of small amounts of *(R*)-enantiomers was confirmed in all quality assurance measures (overlay of the sample-peaks with different amounts of non-racemic standard peaks (*m/z* 74 and *m/z* 87) (see Material and Methods). Hence, it is evident from our data that (*R*)-*a*FAs had been formed during incubations without ILE.

Expressed in total amounts, the concentrations of (newly formed) (*R*)-*a*15:0 was between 1.2 and 4.4 mg/g fat in the rumen fluid (Table 1). Compared to the existing level of *a*15:0 in rumen fluid before the incubation (~30 mg/g fat), this increment was low. As mentioned above, changes in the *a*FA concentration in the course of incubation without ILE differed from year to year (Table 1). Concentrations of *a*FAs were increasing or decreasing with a maximum range of ± 7 mg/g fat during incubation. Taking a slight variation due to inhomogeneity of incubated rumen fluid aliquots into consideration, it can be stated that the concentrations of *a*FAs did not strongly increase in treatments without ILE. This in turn produced strong evidence that the newly-formed (*R*)-*a*15:0 evolved with high stereospecificity because the simultaneous formation of (*S*)-*a*FAs in predominance would have led to an increase in the total *a*FA level. Since racemization of *a*FAs can be excluded (due to the remote position of the stereogenic center and the chemical stability of saturated *a*FAs), the stereospecific formation of (*R*)-*a*FAs points towards an alternative biosynthesis pathway for *a*FAs, possibly due to an altered microbial composition in the incubation solution. It is also evident from our study, that this postulated novel biosynthesis pathway involved utilization of a primer different to L-ILE, which was present in rumen fluid. Previously, small contributions of (*R*)-*a*FAs had been detected in cheese (and especially in the polar lipids) [2]. In retrospective, this finding can also not be explained by the utilization of L-ILE.

**Fig 4:**
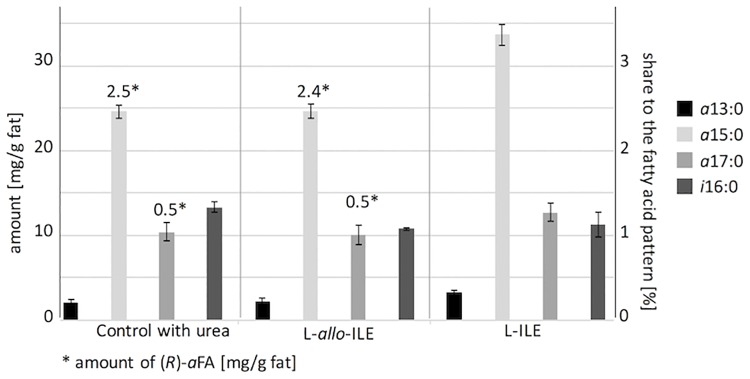
Concentrations [mg/g fat] of *a*13:0, *a*15:0, *a*17:0 and *i*16:0, their contribution to the fatty acid pattern [%] and the concentration of (*R*)-*a*FAs [mg/g fat] including error bars in incubated rumen fluid samples from 2011. Analysis of the concentration of *i*16:0 found no significant differences within the three different treatments (α< 0.01). Significant differences in the concentrations of *a*FAs could be found between, Control with urea” treatment and “L-ILE” with a statistical significance of α< 0.01 while no significant difference could be found between *a*FA concentrations of the control and L-*allo*-ILE treatment (not for α< 0.01 and even not for α< 0.05).

Since the configuration of the amino function on C-2 is decisive for the bioavailability of amino acids in living organisms, L-*allo*-ILE (2*S*, 3*R*-isoleucine, Fig 2c) came into consideration as a possible primer for the formation of (*R*)-*a*FAs [22,42]. This ILE stereoisomer features the required (2*S*)-configuration for being utilized by enzymes for biosynthesis (compare Figs 1 and 2c) [13,41]. In addition, its configuration on C-3 would lead to (*R*)-*a*FAs. In fact, Kaneda observed an increase in (*S*)-configurated *a*15:0 and *a*17:0 concentration compared to controls during incubation of *B*. *subtilis* with L-*allo*-ILE albeit to a lesser degree compared with L-ILE [13]. Our rumen fluid incubations with L-*allo*-ILE resulted in the generation of small amounts of (*R*)-*a*FAs (*a*15:0-ME with ee = 80% and *a*17:0-ME with ee = 90%; Table 2). However, the *a*FA concentration in rumen fluid fat did not differ significantly (α < 0.05) in comparison to the control experiment (Fig 4). Two explanations could explain this observation: either (i) a minute amount of L-*allo*-ILE was utilized to generate (*R*)-*a*FAs or (ii) L-*allo*-ILE was not utilized at all and the observed (*R*)-*a*FAs originated from the currently unknown source as observed in the control samples.

Literature experiments produced evidence for *in vivo* conversion of L-ILE into L-*allo*-ILE via enantiomerization of (*S*)-2-keto-3-methylvaleric acid (KMVA) into (*R*)-KMVA [43,44]. However, different mutants of *Bacillus* verified the exclusive use of (*S*)-KMVA by branch-chained 2-ketoacid dehydrogenase [8,9,13,41]. The stereospecificity of this reaction inhibited the formation of (2*R*)-methylbutyryl-CoA from (*R*)-KMVA and excluded the formation of (*R*)-*a*FAs from L-*allo*-ILE via this mechanism [8,9,13,41]. In agreement with that and in contrast to L-ILE, incubation with L-*allo*-ILE, did neither increase the share of *a*FAs (*a*13:0, *a*15:0 and *a*17:0) nor of *i*16:0 (Fig 4). These aspects strongly support explanation (ii), i.e. that L-*allo*-ILE was not utilized by the rumen bacteria to generate (*R*)-*a*FAs.

## Conclusion

The presented incubation experiments with rumen fluid verified the significant formation of (*R*)-*a*FAs. We further produced strong evidence for the existence of a different primer in rumen fluid which can be utilized for the biosynthesis of (*R*)-*a*FAs. Conceivable primers are short chained chiral compounds with a methyl branch on the antepenultimate carbon. The corresponding primer of (*R*)-*a*FAs could either be (*R*)-enantiopure or the consequence of its partial enantiomerization during biosynthesis. Possible primers for this pathway could be 2-methyl butanol or 2-methyl butanal. A second alternative route could be the elongation of 4-methyl hexanoic acid. Recently, it was found that 4-methyl-branched fatty acids in sheep and goat are (*R*)-enantiopure [45]. One intermediate compound of this biosynthesis pathway would be 4-methyl hexanoic acid which is both belonging to the family of 4-alkyl-branched fatty acids (dominance of (*R)*-enantiomers) and to the family of *anteiso*-fatty acids (dominance of (*S)*-enantiomers). Finally, methylation of an unsaturated acid (or intermediate), e.g. mediated by *S*-adenosyl methionine could be a third biosynthesis route for (*R*)-*a*FAs. Due to the huge variety of microorganisms in rumen fluid and its chemical complexity our experimental setup is not suited to elucidate the prerequisites and conditions for an alternative biosynthesis of *a*FAs, i.e. without the utilization of ILE. More biochemically-based research will be required to identify primer(s) and microorganisms involved in the formation of (*R*)-*a*FAs. At this point it appears that there is a driving force in the rumen microbiome to generate *a*FAs. If ILE is not available and the classic biosynthesis pathway cannot occur, another route is gone. The previous detection of (*R*)-*a*FAs in cheese and fish [2,5] indicates that this alternative formation of *a*FAs is widespread in nature.

## Supporting Information

S1 Table Overview of different incubation treatments performed in different years with substances and amounts [mg] added. (DOCX)

S2 Table Concentrations (mg/g fat) of *iso*-fatty acids in rumen fluid before (unincubated) and after incubation with carbohydrates only or with carbohydrates and urea, L-ILE or DL-ILE. (DOCX)
