# Supplementary material for: Unexpected Formation of Low Amounts of (R)-Configurated anteiso-Fatty Acids in Rumen Fluid Experiments
Source: PLoS One. 2017 Jan 27;12(1):e0170788. doi: 10.1371/journal.pone.0170788 (PMC5271357; doi:10.1371/journal.pone.0170788)
Supplement: S1 Table — (DOCX) [file pone.0170788.s001.docx]

| treatment | CHO-mixture | urea | L-ILE | D/L-ILE | L-*allo*-ILE | Year(s) |
| --- | --- | --- | --- | --- | --- | --- |
|  | [mg] | [mg] | [mg] | [mg] | [mg] |  |
| 1 | ~ 200 |  | ~13 |  |  | 2010,2011,2015 |
| 2 | ~ 200 |  |  | ~13 |  | 2010,2011,2015 |
| 3 | ~ 200 |  |  |  |  | 2010,2011,2015 |
| 4 | ~ 200 | ~6 |  |  |  | 2010,2011,2015 |
| 5 | ~ 200 | 7.4 |  |  | ~12 | 2011 |
| 6 | ~ 200 | 7.4 | ~12 |  |  | 2011 |
| 7 | ~200 | 10.4 |  |  |  | 2011 |

**S1 Table. Overview of different incubation treatments performed in different years with substances and amounts [mg] added.**
