# Supplementary material for: Unexpected Formation of Low Amounts of (R)-Configurated anteiso-Fatty Acids in Rumen Fluid Experiments
Source: PLoS One. 2017 Jan 27;12(1):e0170788. doi: 10.1371/journal.pone.0170788 (PMC5271357; doi:10.1371/journal.pone.0170788)
Supplement: S2 Table — (DOCX) [file pone.0170788.s002.docx]

**S2 Table. Concentrations (mg/g fat) of *iso*-fatty acids in rumen fluid before (unincubated) and after incubation with carbohydrates only or with carbohydrates and urea, L-ILE or DL-ILE**

|  | **Carbohydrates only** | | | **Carbohydrates and urea** | | | **Carbohydrates and L-ILE** | | | **Carbohydrates and DL-ILE** | | | **Unincubated rumen fluid** | |
| --- | --- | --- | --- | --- | --- | --- | --- | --- | --- | --- | --- | --- | --- | --- |
|  | **2015** | **2011** | **2010** | **2015** | **2011** | **2010** | **2015** | **2011** | **2010** | **2015** | **2011** | **2010** | **2015** | **2011** |
| **mg/g fat** | | | | | | | | | | | | | | |
| *i*13:0 | 2.90 | 2.20 | 1.00 | 1.99 | 2.80 | 1.00 | 2.73 | 2.20 | 1.00 | 2.74 | 2.10 | 1.00 | 1.90 | 2.80 |
| *i*14:0 | 15.1 | 6.50 | 9.50 | 9.86 | 9.00 | 12.5 | 6.20 | 3.60 | 9.50 | 12.2 | 4.80 | 5.00 | 5.40 | 6.00 |
| *i*15:0 | 17.1 | 11.2 | 10.0 | 13.4 | 13.1 | 10.0 | 16.0 | 11.4 | 10.0 | 16.1 | 12.2 | 15.1 | 3.20 | 11.0 |
| *i*16:0 | 19.07 | 14.4 | 16.1 | 11.6 | 16.7 | 19.5 | 11.5 | 8.30 | 16.2 | 19.8 | 11.0 | 22.5 | 15.2 | 10.1 |
| *i*17:0 | 7.08 | 7.70 | 6.50 | 4.81 | 7.30 | 4.50 | 5.35 | 5.80 | 5.50 | 5.06 | 7.00 | 6.50 | 9.40 | 7.70 |
| *i*18:0 | 0.68 | 0.60 | n.d. | 0.45 | 1.00 | n.d. | 0.66 | 0.80 | n.d. | 0.56 | 0.90 | n.d. | 1.20 | 0.60 |
